# Anesthetic modulations dissociate neuroelectric characteristics between sensory-evoked and spontaneous activities across bilateral rat somatosensory cortical laminae

**DOI:** 10.1038/s41598-022-13759-0

**Published:** 2022-07-08

**Authors:** Kwangyeol Baek, Chae Ri Park, Siwan Jang, Woo Hyun Shim, Young Ro Kim

**Affiliations:** 1 School of Biomedical Convergence Engineering, Pusan National University, Busan, Republic of Korea; 2 Department of Medical Science, Asan Medical Institute of Convergence Science and Technology, Asan Medical Center, University of Ulsan College of Medicine, Seoul, Republic of Korea; 3 Washington University in St. Louis, St. Louis, MO USA; 4 Department of Radiology, Asan Medical Center, College of Medicine, University of Ulsan, Ulsan, South Korea; 5 Athinoula A. Martinos Center for Biomedical Imaging, Massachusetts General Hospital, Charlestown, MA USA; 6 Department of Radiology, Harvard Medical School, Boston, MA USA

**Keywords:** Neuroscience, Physiology

## Abstract

Spontaneous neural activity has been widely adopted to construct functional connectivity (FC) amongst distant brain regions. Although informative, the functional role and signaling mechanism of the resting state FC are not intuitive as those in stimulus/task-evoked activity. In order to bridge the gap, we investigated anesthetic modulation of both resting-state and sensory-evoked activities. We used two well-studied GABAergic anesthetics of varying dose (isoflurane: 0.5–2.0% and α-chloralose: 30 and 60 mg/kg∙h) and recorded changes in electrophysiology using a pair of laminar electrode arrays that encompass the entire depth of the bilateral somatosensory cortices (S1fl) in rats. Specifically, the study focused to describe how varying anesthesia conditions affect the resting state activities and resultant FC between bilateral hemispheres in comparison to those obtained by evoked responses. As results, isoflurane decreased the amplitude of evoked responses in a dose-dependent manner mostly due to the habituation of repetitive responses. However, α-chloralose rather intensified the amplitude without exhibiting habituation. No such diverging trend was observed for the spontaneous activity, in which both anesthetics increased the signal power. For α-chloralose, overall FC was similar to that obtained with the lowest dose of isoflurane at 0.5% while higher doses of isoflurane displayed increased FC. Interestingly, only α-chloralose elicited relatively much greater increases in the ipsi-stimulus evoked response (i.e., in S1fl ipsilateral to the stimulated forelimb) than those associated with the contra-stimulus response, suggesting enhanced neuronal excitability. Taken together, the findings demonstrate modulation of the FC profiles by anesthesia is highly non-linear, possibly with a distinct underlying mechanism that affects either resting state or evoked activities differently. Further, the current study warrants thorough investigation of the basal neuronal states prior to the interpretation of resting state FC and evoked activities for accurate understanding of neural signal processing and circuitry.

## Introduction

The brain works as a large network consisting of multiple functional modules and massively interconnected populations of distinctive neurons^1^. Typical brain activity can be described as the neuronal activation elicited by task/stimulus and propagation of neuroelectric and cerebrovascular events across the functionally related brain regions^2,3^. In conjunction with underlying anatomical architecture, the neuronal networks and their functional connections have been assessed by monitoring task-driven and/or stimulus-elicited activities and ensuing interactions. On the other hand, the brain also exhibits abundant spontaneous activities devoid of any external stimuli or tasks, which has been demonstrated rigorously in both human subjects and animal models^4–7^. The acquisition and application of spontaneous activities have gained an explosive popularity in recent years for the advancement of signal acquisition techniques and wide availability of analysis options. In particular, the statistical dependence among neuronal signal fluctuations obtained during resting (or non-task/stimulus) state has been extensively investigated and used for construction of functional connectivity (FC) maps^8^.

Upon phenotypic inspection of the resting state FC, a number of studies have related the altered FC integrity with disease progression and/or level of task performance^9–13^. Despite the growing interest in the phenomenon and feasibility of application, the role and signaling mechanism of spontaneous brain activity are not understood well. To date, the FC based on spontaneous activity is only hypothesized to reveal brain areas with a shared function in information processing but very much limited in other practical purposes^6^. On the other hand, evoked responses have been used to construct hypothetical neural networks based on the well-defined functional segregation of brain activities^14–18^. In these regards, the patterns of such effective connectivity have been assessed using either directed interactions of evoked responses or the spontaneous activities, the results from which were occasionally compared with each other^19,20^. The functional networks derived from these different strategies revealed similarity, roughly in prediction of each other’s outcome^21^. However, it is not yet clear how the neural processing of tasks is neurophysiologically related with the functional circuits in spontaneous brain activities^19,20,22,23^.

Previous evidences from numerous animal model studies demonstrated that spontaneous electrophysiological activities form the basis of the observed resting state FC^24–28^. In rats, delta band activity was implicated with the spontaneous BOLD fluctuations responsible for the FC across the bilateral somatosensory cortices^24^. Slow modulation of the spiking or gamma band activities was associated with the resting state BOLD FC in the monkey visual cortex^26^. However, these animal studies often involved an altered state of neurophysiology, influenced mainly by the use of anesthetics and sedatives^29^. In this regard, a slew of animal studies demonstrated potent, dose-dependent effects of anesthetics and subsequent changes in the construction of FC as well as in the evoked responses^18,23,30,31^. Since the clinical application of resting-state FC requires a parallel evaluation that links animal and human results, it is imperative to investigate the effects of such anesthetics on the spontaneous activity and sensory processing at the neuronal function level.

Based on our previous findings in the interhemispheric FC and sensory-evoked activation in the rat brain^32^, the experiments in this study were designed to modulate both evoked response and spontaneous activity by varying the levels and types of anesthetics. For this purpose, two commonly used anesthetics isoflurane and α-chloralose were used, the effects of which have been well documented in numerous neuroimaging and physiology studies in rodent model^33,34^. In brief, isoflurane is known to reduce junctional conductance via the potassium channel and binding to GABA, glutamate, and glycine receptors^29,35^ whereas α-chloralose works as a sedative, characterized by potent effects at the inhibitory GABA type A (GABA_A_) receptors^36^. Commonly described effects of GABA_A_ on the resting state electrophysiology include alteration of cortical slow wave (0.1–1 Hz) with alternating periods of silent/DOWN- and active/UP-states, which progressively evolve into burst-suppression mode with increasing depth of anesthesia^29,37^. In association of resting state activities with evoked responses, previous studies indicated that peripheral stimulation is known to induce larger cortical responses when the cortex is in the DOWN (i.e., hyperpolarized) state compared to the UP state^29,38,39^. However, the variable anesthetic influences on the neuronal excitation and processing of evoked-response and spontaneous activities are still speculative and require further investigation.

The current study was designed to compare electrophysiological outcomes acquired under the varying levels of anesthetic influence with a particular focus to determine the relationship between the profiles of evoked neuronal excitation and spontaneous events. We tested whether spontaneous and sensory-evoked activities are similarly affected by varying anesthetic conditions to reveal if both share the same neural circuit and mechanism. In this regard, the experiments focused on the comprehensive comparison of electrophysiological traits acquired under variable anesthesia conditions. Specifically, electrophysiological profiles of the evoked responses to electrical forelimb stimulation and resting state interlaminar and inter-hemispheric FC were acquired in the bilateral somatosensory cortices of rats, which is one of the most studied functional networks in animal studies paralleling human findings^4,24,40^. The study aimed to demonstrate how two different types of anesthetics affect the cortical excitability and neural dynamics, and to explore a possible link between the modulated evoked and spontaneous brain activities.

## Results

### Spontaneous LFP activity along the cortical layers is distinctively altered by anesthetics

**Figure 1:**
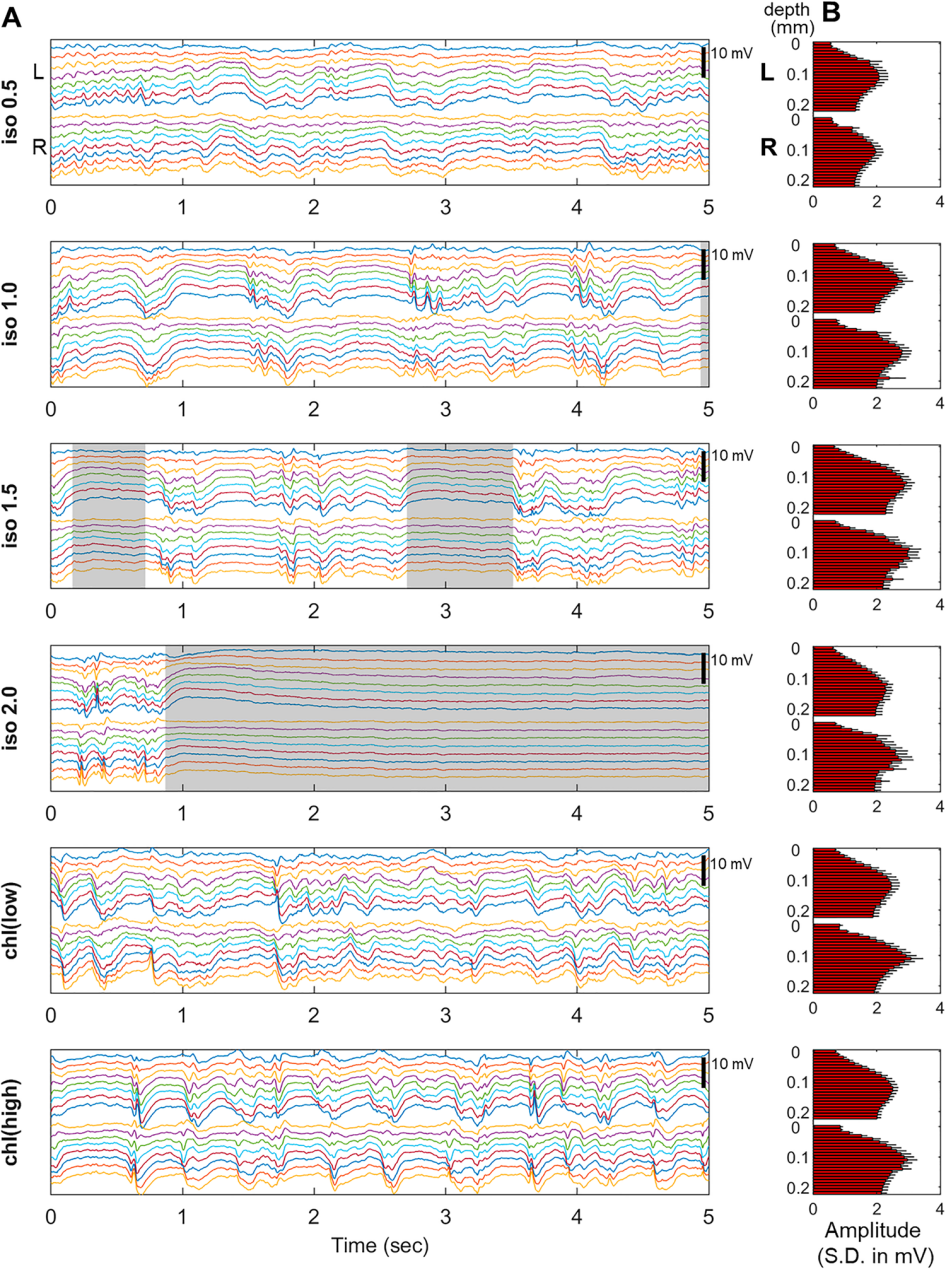
Spontaneous LFP activity across different anesthesia conditions for left (L) and right (R) S1fl. (**A**) An example of LFP recordings from a representative animal using a pair of laminar electrodes (every three channels are shown, i.e. depth of 0.1, 0.4, 0.7, 1.0, 1.3, 1.6, 1.9 and 2.2 mm). Shaded areas indicate suppression periods. (**B**) The amplitudes of spontaneous LFP fluctuations (Standard deviation in spontaneous LFP activity, in mV) along the cortical depth. Group mean ± S.E. (n = 8).

We acquired spontaneous LFP activity in the forelimb region of the bilateral primary somatosensory cortices (S1fl) using two linear multi-electrode arrays (with 23 contacts spaced by 100 μm). Examples of spontaneous LFP activity recordings from a rat under different anesthestic conditions are shown in Fig. 1. As the isoflurane dose increased (1.0% or higher), apparent burst-suppression pattern emerged, in which a period of suppression (defined by LFP fluctuation < 0.1 mV/ms for a period longer than 500 ms) was followed by bursts of large LFP activities. For anesthesia with α-chloralose, such burst-suppression pattern was not observed.

Suppression periods during isoflurane anesthesia were quantified as shown in Table 1, in which no suppression was observed for 0.5% isoflurane and any α-chloralose conditions. In general, the average duration of the suppression periods (inter-burst interval) increased as the isoflurane dose increased (p < 0.05, Friedman test). The suppression ratio (the ratio between the total suppression period and the total recording time) also increased as the dose of isoflurane increased, indicating the proportional reduction of the burst periods (p < 0.0001, Friedman test).

**Table 1:** Burst-suppression characteristics were observed under anesthesia with isoflurane (1.0% or higher) but not with α-chloralose.

Anesthesia	Suppression ratio, Mean ± S.D	Suppression duration (ms), Mean ± S.D
isoflurane 0.5%	0.0 ± 0.0	0.0 ± 0.0
isoflurane 1.0%	0.068 ± 0.138	**703 ± 170 **^**a**^
isoflurane 1.5%	0.206 ± 0.264	**992 ± 421 **^**a**^
isoflurane 2.0%	**0.589 ± 0.227 **^**c**^	**1728 ± 802 **^**b**^

Suppression periods were defined as periods in which LFP fluctuations were less than 0.1 mV/ms for a period of 500 ms or longer, while suppression ratio is the ratio of total suppression period to total recording time. (n = 8).

^a^p < 0.01 for isoflurane 1.0% > isoflurane 0.5% and isoflurane 1.5% > isoflurane 0.5% (post-hoc Tukey’s test).

^b^p < 0.01 for isoflurane 2.0% > isoflurane 1.5%, and p < 0.001 for isoflurane 2.0% > isoflurane 1.0% (post-hoc Tukey’s test). p < 0.001 for isoflurane 2.0% > isoflurane 0.5%.

^c^p < 0.05 for isoflurane 2.0% > isoflurane 1.5% and isoflurane 2.0% > isoflurane 1.0% (post-hoc Tukey’s test). p < 0.001 for isoflurane 2.0% > isoflurane 0.5%.

**Figure 2:**
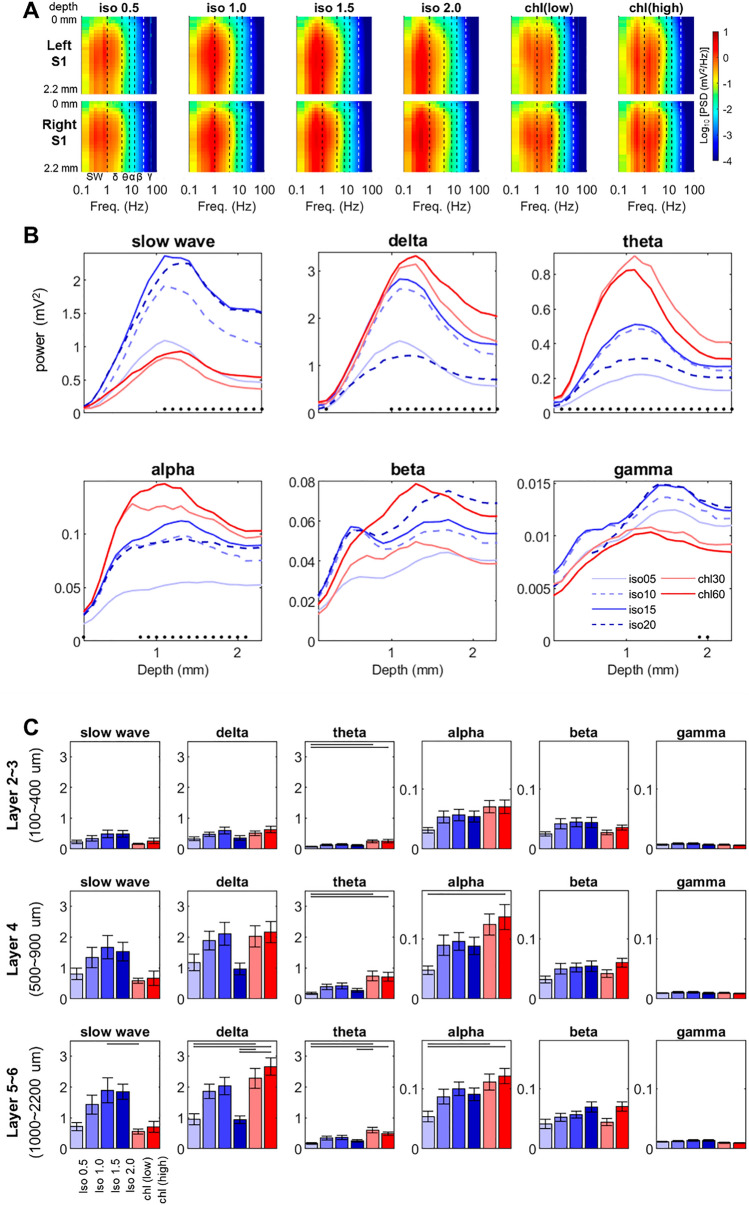
Spectral power of spontaneous LFP activity across different anesthesia conditions. (**A**) Power spectral density (PSD, in mV^2^/Hz) along the cortical depth. (**B**) Comparison of band power distribution along the cortical depth. Left and right S1fl data were combined in this plot. Black dots indicate significant difference across anesthesia conditions (p < 0.05, Friedman test). (**C**) Post-hoc comparisons on the average band power in the supragranular (layer 2 ~ 3), the granular (layer 4) and the infragranular layer (layer 5 ~ 6) (p < 0.05, Tukey’s test). Note that y-axis scale is 0 ~ 3.5 mV^2^ for slow wave, delta and theta bands, and 0 ~ 0.18 mV^2^ for alpha, beta and gamma bands. Slow wave: 0.1 ~ 1 Hz, Delta: 1 ~ 4 Hz, Theta: 4 ~ 8 Hz, Alpha: 8 ~ 13 Hz, Beta: 13 ~ 30 Hz, Gamma: 30 ~ 100 Hz. Horizontal bar: p < 0.05 in post-hoc Tukey’s test. (n = 16; 8 rats × 2 hemispheres).

Spectral power analysis revealed that the anesthesia-induced alteration of LFP power occurred throughout the entire cortical depth (see Fig. 2). Both the peak power and the corresponding frequency (i.e., peak frequency) tend to be dependent on the anesthetic type and dose as well as on the cortical depth. As the dose of isoflurane increased (> 1.0%), the peak frequency found in the power spectral density decreased (see Fig. S1). On the other hand, α-chloralose induced multiple frequency peaks in the spectral power for slow waves and delta band. In the frequency range above the peak frequency, the spectral power decreased as the frequency increased for each anesthetic condition as shown in Fig. 2A and S1. The appearance is in accordance with the 1/f^N^ power spectral relation which has been consistently reported in previous studies^41^. Slow waves, delta and theta bands exhibited much larger spectral power than higher frequency bands (i.e. alpha, beta and gamma bands) in all anesthetic conditions (note the y-axis scales in Fig. 2B,C).

As shown in Fig. 2B,C, the spectral band powers were smallest at 0.5% isoflurane for most frequency bands, and readily increased with the higher dose of isoflurane. In isoflurane anesthesia, delta band and slow wave activities were dominant while slow wave activity tended to increase as the dose of isoflurane increased. For isoflurane, the dose-dependent increases in the band power saturated reaching the maximum around 1.5% (except for beta band). As the dose of isoflurane increased from 1.5 to 2.0%, the delta and theta band powers decreased likely due to the emergence of strong suppression. The trend changed for α-chloralose, in which the delta band activity was dominant, but slow wave activity was much smaller than isoflurane anesthesia. Moreover, the theta band activity (4 ~ 8 Hz) was significantly higher in α-chloralose conditions compared to 0.5% isoflurane throughout the entire cortical layers (Fig. 2B,C). In general, α-chloralose increased the theta band activity maximally at the depth around 1100 μm (upper layer 5) as shown in Fig. 2B. Notable increases of the delta (1 ~ 4 Hz) and alpha band powers (8 ~ 13 Hz) were also observed for α-chloralose anesthesia when compared to the 0.5% isoflurane condition (see Fig. 2B,C). For both beta (13 ~ 30 Hz) and gamma (30 ~ 100 Hz) band powers, no significant differences were found among any conscious levels (Fig. 2C).

In summary, different types and doses of anesthetics modulated the spontaneous neural activity in both spectral power distribution and spatial profile of LFP activities along the cortical depth. Burst-suppression pattern was observed for the anesthesia with isoflurane doses greater than 1.0% but not for α-chloralose and 0.5% isoflurane. Compared to the weak sedation state with a minimal dose of isoflurane (i.e. isoflurane 0.5%), high doses of isoflurane substantially increased the slow wave activity and enhanced the power particularly in deep cortical layers (i.e., layer 5 ~ 6).

### Functional connectivity shows distinctive laminar pattern depending on the types of anesthetics

**Figure 3:**
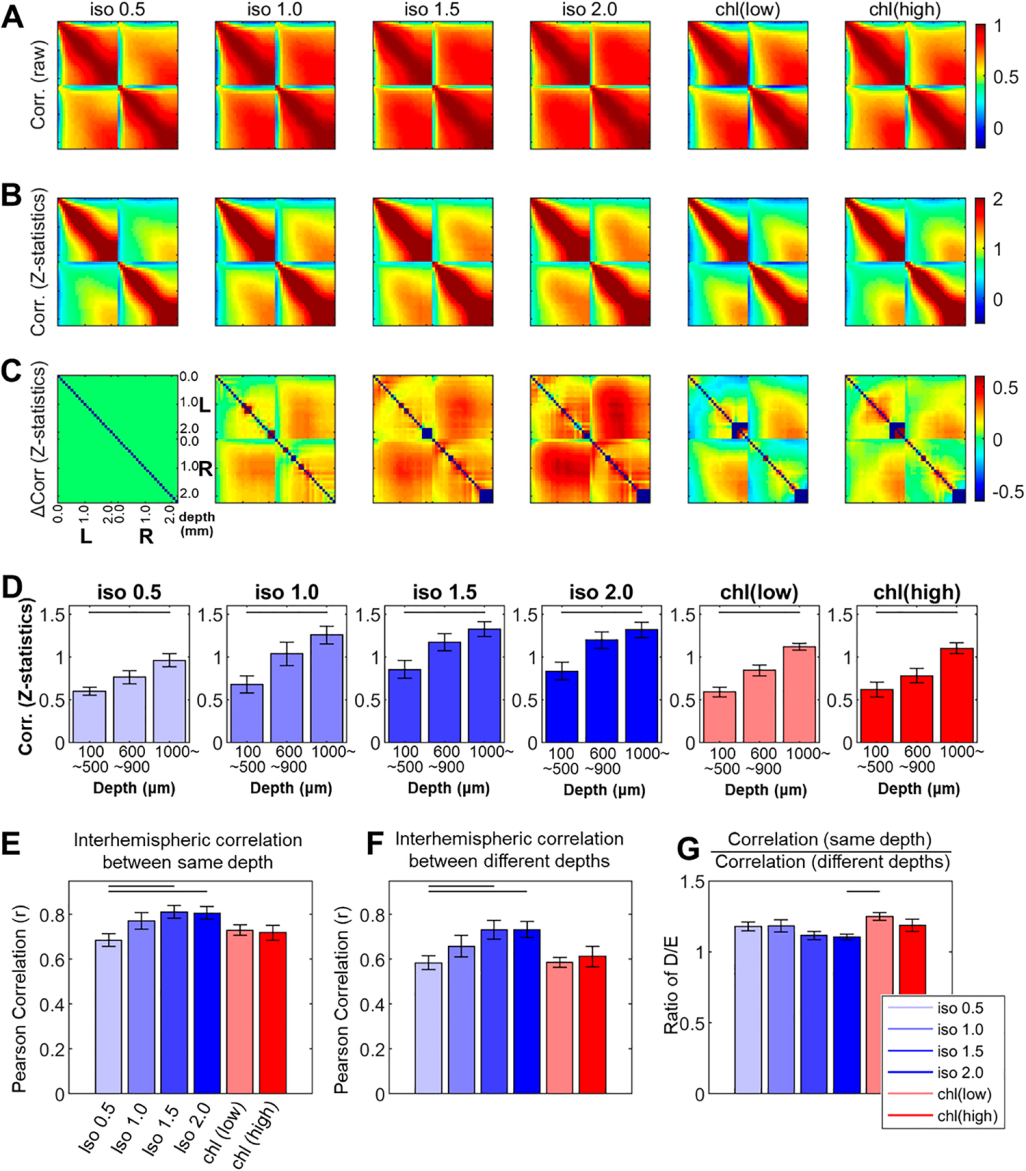
Laminar correlation in spontaneous LFP activity along cortical depth (0.0 ~ 2.2 mm) across different anesthesia conditions (Group mean, n = 8). (**A**) Pearson’s correlation coefficient *r* across spontaneous LFP activity in bilateral S1fl. (**B**) Z-statistics calculated by Fisher transformation. (**C**) Difference in z-statistics between isoflurane 0.5% and other anesthesia conditions. High dose of isoflurane increased the diffused correlation centered around middle layers, but α-choloralose (low dose, in particular) strengthened interhemispheric correlation in a layer-specific pattern. (**D**) Inter-hemispheric correlation (z-statistics) at the supragranular, the granular and the infragranular layers. (**E**) Average inter-hemispheric correlation between left and right S1fl at same depth. (**F**) Average inter-hemispheric correlation between left and right S1fl at different depths. (**G**) Layer-specificity in the interhemispheric correlation estimated by ratio of E (same depth) and F (different depths). Horizontal bar: p < 0.05 in post-hoc Tukey’s test.

As shown in Fig. 3A, laminar FC was constructed within the unilateral cortex and between bilateral cortices (i.e., interlaminar and inter-hemispheric correlations) using zero-lag Pearson’s correlation coefficient *r*. Additionally, z-statistic was calculated from *r* using Fisher transformation (See Fig. 3B,C) for better comparison of FC between anesthetic conditions.

Layer-specific patterns were apparent for both interlaminar- and inter-hemispherical correlations obtained in the minimal dose of isoflurane (0.5%) condition, in which no burst-suppression pattern was observed (see Table 1). The interlaminar correlation within the unilateral cortex at 0.5% isoflurane revealed higher correlation values between cortical depths at 300–900 μm and at 1300–2300 μm than the rest of layers. The inter-hemispheric correlation was grossly characterized by (i) weak correlations between upper layers and middle/deep layers and (ii) relatively high correlations between deep layers (Fig. 3D). For higher isoflurane doses, isoflurane induced less specific pattern of inter-hemispheric correlation in spontaneous LFP activity along the cortical depth (Fig. 3A), which is likely associated with the burst-suppression activity observed in isoflurane doses of 1.0% or higher (See Fig. S2 for the laminar correlation patterns within the burst periods or suppression periods). In general, higher doses of isoflurane (1.5% and 2.0% isoflurane) tend to increase the interhemispheric correlation (FC) at the same depth as well as different depths (See Fig. 3E,F). The globally synchronized activity readily increased as the dose increased, which resulted in the diffused global correlation along the cortical depth (Fig. 3C). However, z-statistic analysis (Fig. 3B) revealed that a layer-specific pattern was not fully disrupted and also that the inter-hemispheric correlations in upper/middle layers likely increased in a dose-dependent manner.

It is notable that α-chloralose also exhibited the layer-specific organization of interhemispheric correlation similar to that exhibited under 0.5% isoflurane. Upon visual inspection of spatial distribution, α-chloralose anesthesia rather increased interhemispheric correlation for both doses (30 and 60 mg/kg∙h) in a layer-specific manner compared to 0.5% isoflurane and in contrast to higher doses of isoflurane (Fig. 3C). However, quantitative analyses failed to confirm the statistical significance of such increases as shown in Fig. 3E. In addition, in order to compare the level of layer-specificity, we calculated the ratio of the average interhemispheric correlation at the same depth as the average of those between the different depths (Fig. 3G). In general, this ratio (i.e., layer-specificity) was greater than 1 for all anesthetic conditions and decreased as the dose increased for both isoflurane and α-chloralose. This layer-specificity ratio was modulated across the anesthetic conditions, but a statistically significant difference was only found between α-chloralose (30 mg/kg.hr) and isoflurane 2.0% conditions (Fig. 3G; p < 0.05 in a post-hoc Tukey’s test), showing relative enhancement by the low dose α-chloralose. We also compared the average correlation between cortical layers (i.e. supragranular, granular and infragranular layers). High dose of isoflurane (1.5% and 2.0%) generally increased inter-hemispheric correlation across the granular and the infragranular layers. (See Fig. S3 for details).

**Figure 4:**
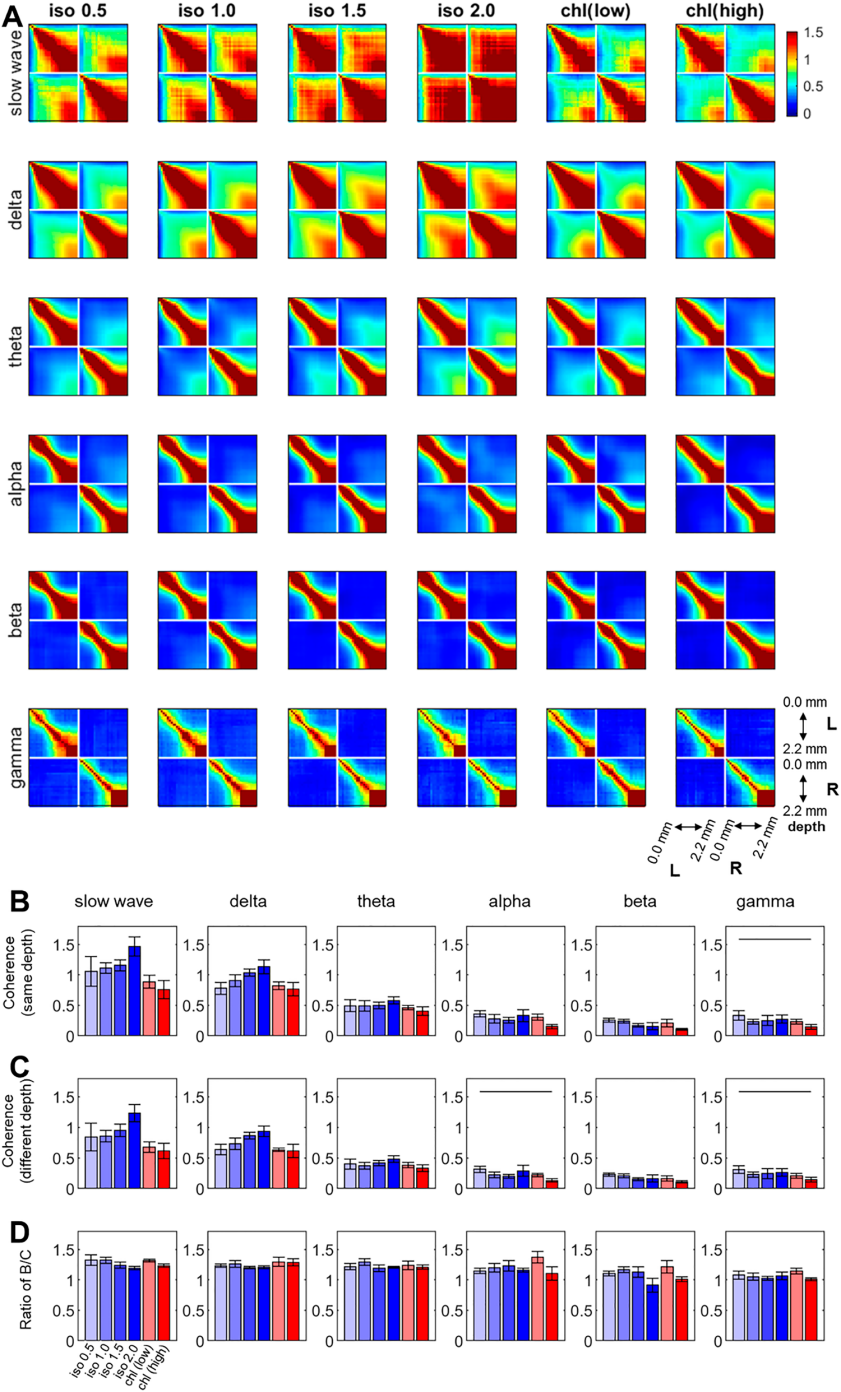
Band coherences of spontaneous LFP activities across varying anesthesia conditions. (**A**) Z-statistics for band coherence using Fisher transformation. (**B**) Average z-statistics at same depth. (**C**) Average z-statistics at different depths. (**D**) Layer-specificity of inter-hemispheric coherences (Ratio of B/C). Horizontal bar: p < 0.05 in post-hoc Tukey’s test. (n = 8. Group average).

As shown in Fig. 4, interlaminar and inter-hemispheric coherences were evaluated across various frequency bands. For 0.5% isoflurane condition, the interlaminar correlation of low frequency oscillations (slow wave, delta and theta bands) within the unilateral cortex were spatially diffused across the cortical depth. In contrast, other high frequency oscillations (alpha, beta and gamma bands) showed a distinctive boundary at ~ 1000 μm (the upper boundary of layer 5) in the interlaminar correlation, spatially differentiating neural populations. Inter-hemispheric coherences tended to be more pronounced for low frequency oscillations (i.e., slow wave, delta, theta and alpha bands) than other high frequency bands across varying anesthesia conditions. Such inter-hemispheric coherences in beta and gamma bands were relatively low and had much less contribution to the total interhemispheric FC than other frequency band activities (e.g., approximately 20% of slow wave). Spatially nonspecific global correlation manifested mainly in the slow wave and delta band as the isoflurane dose increased from 0.5 to 2.0%, although a post-hoc test failed to confirm any statistically significant change (See Fig. 4B,C). In addition, the interhemispheric coherences in alpha and gamma bands were significantly lower for the high dose α-chloralose when compared to the 0.5% isoflurane. For both anesthetics, no noticeable changes in the layer-specificity of coherences were observed (Fig. 4D).

### Evoked response (averaged over a stimulus-train) is reduced by isoflurane but elevated by α-chloralose

**Figure 5:**
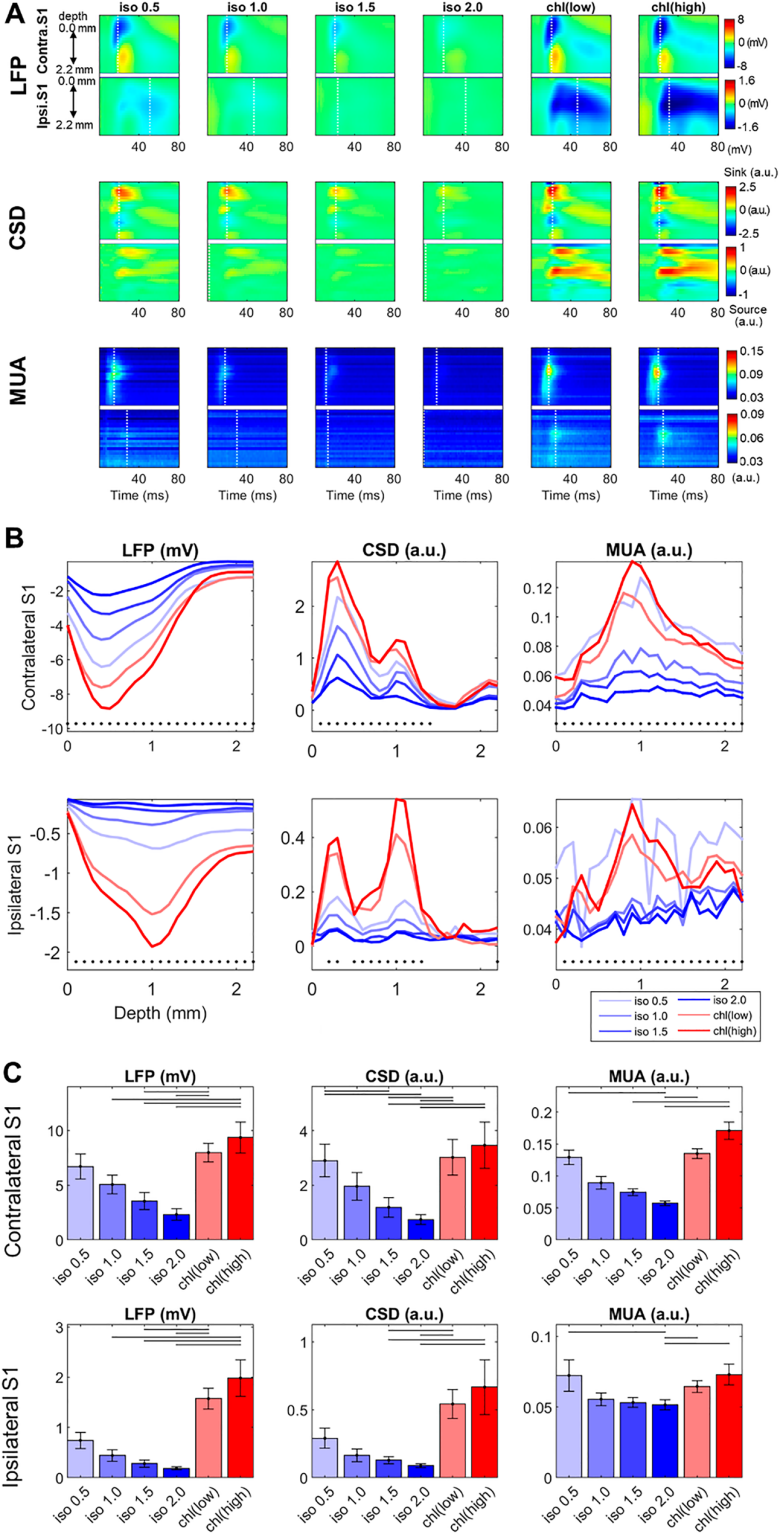
Averaged evoked response to electrical forepaw stimuli (1.0 mA, 0.3 μs duration, 3 Hz for 2 s). (**A**) Spatiotemporal pattern of the sensory-evoked response in the bilateral S1fl. White dotted line: time point with the peak amplitude. (**B**) Peak amplitude in sensory-evoked response in LFP, CSD and MUA (within 0 ~ 50 ms after the forepaw stimulation). Black dots: p < 0.05 in Friedman test. (**C**) Post-hoc comparisons across anesthetic conditions (p < 0.05, Tukey’s test). The highest absolute value in peak amplitudes along the cortical depth were compared. Horizontal bar: p < 0.05 in post-hoc Tukey’s test. (n = 16; 8 rats × 2 hemispheres).

The averaged sensory-evoked responses from combined left and right S1fl results are shown in Fig. 5 (see Fig. S4 for line traces and Fig. S5 for separate left and right S1fl results). Under sedation with isoflurane 0.5%, electrical forelimb stimulation induced robust sensory-evoked responses in the contra-stimulus (i.e., contralateral to the stimulated forelimb) S1fl region as shown in Fig. 5, in which the peak LFP response was -6.04 ± 2.31 mV (Mean ± S.D.) at the cortical depth of 400 ~ 500 μm. The ipsi-stimulus LFP responses were delayed by 6 ~ 8 ms with its peak much smaller (0.78 ± 0.50 mV) and in the deeper location (depth of ~ 1000 μm) than the contra-stimulus counterparts (Fig. 5 and S4), which is in accordance with the previous studies^32,42^. Current source density analysis (CSD) revealed two primary current sinks in the contra-stimulus S1fl where an early current sink at the cortical depth of ~ 1000 μm (the boundary between layers 4 and 5) was followed by a larger current sink at the cortical depth of 300 ~ 400 μm (layer 2/3) as shown in Fig. 5A and S4A^32^. The peak CSD response in the ipsi-stimulus S1fl was less than 10% in amplitude compared to that of the contra-stimulus response with an initial response appearing at the depth of ~ 1100 μm followed up by a trace at ~ 300 μm. At 0.5% isoflurane, the multi-unit activity (MUA) response was focalized at the cortical depth of ~ 1000 μm in the contra-stimulus S1fl while no profound ipsi-stimulus MUA response peaks were observed (Fig. 5A).

In general, higher doses of isoflurane reduced the contra-stimulus response amplitude in a dose-dependent manner, in which the peak amplitudes in the contra-stimulus evoked LFP response decreased to 76%, 48%, and 34% of that quantified at 0.5% for 1.0%, 1.5%, and 2.0% isoflurane, respectively. In comparison with 0.5% isoflurane, the contra-stimulus CSD amplitudes also dropped to 73%, 40% and 27% while the MUA response also diminished to 64%, 53%, and 45% for isoflurane doses of 1.0%, 1.5%, and 2.0%, respectively. A Friedman test revealed statistically significant dose-dependent effects of isoflurane in the contra-stimulus S1fl response (i.e., across 1.0%, 1.5% and 2.0%), where all p values were less than 0.001 in LFP, CSD and MUA. As was in contra-stimulus S1fl, the ipsi-stimulus S1fl response was affected by isoflurane in a dose-dependent manner, in which the ipsi-stimulus response amplitude generally decreased by isoflurane in all variables (all p < 0.001 in LFP, CSD and MUA).

Interestingly, α-chloralose showed stronger sensory-evoked responses in the bilateral S1fl regions compared to isoflurane anesthesia. The peak LFP responses in the contra-stimulus S1fl increased by 32% and 55% of that measured with 0.5% isoflurane for α-chloralose 30 and 60 mg/kg∙hr conditions, respectively and with statistical significance (p < 0.05, Friedman test with a post-hoc Tukey’s test) when compared to higher doses of isoflurane (1.0%). Similarly, statistically significant increases in the peak CSD and MUA were also observed. The dual peak pattern of contra-stimulus CSD found in the 0.5% isoflurane (at the cortical depths of ~ 400 and ~ 1100 μm) was well preserved with α-chloralose with roughly proportional enhancements along the cortical depth. In the ipsi-stimulus S1fl, evoked responses were even more drastically enhanced by α-chloralose than contra-stimulus. Compared to the 0.5% isoflurane condition, the peak LFP amplitude in ipsi-stimulus S1fl increased by 116% and 168% for α-chloralose at 30 and 60 mg/kg∙h, respectively (p < 0.01 for both) while the peak CSD responses increased by 98% and 143%, respectively (p < 0.01 and p < 0.05, respectively). In general, despite such striking changes in magnitude, spatiotemporal characteristics of the CSD responses under α-chloralose did not alter compared to those from isoflurane. In contrast to LFP and CSD, the peak MUA responses did not enhance much compared to those acquired from isoflurane anesthesia. In conclusion, α-chloralose substantially enhanced the sensory-evoked responses in bilateral S1fl regions with particularly strong increases in the ipsi-stimulus S1fl responses.

### Habituation of repeated evoked responses is differently altered

**Figure 6:**
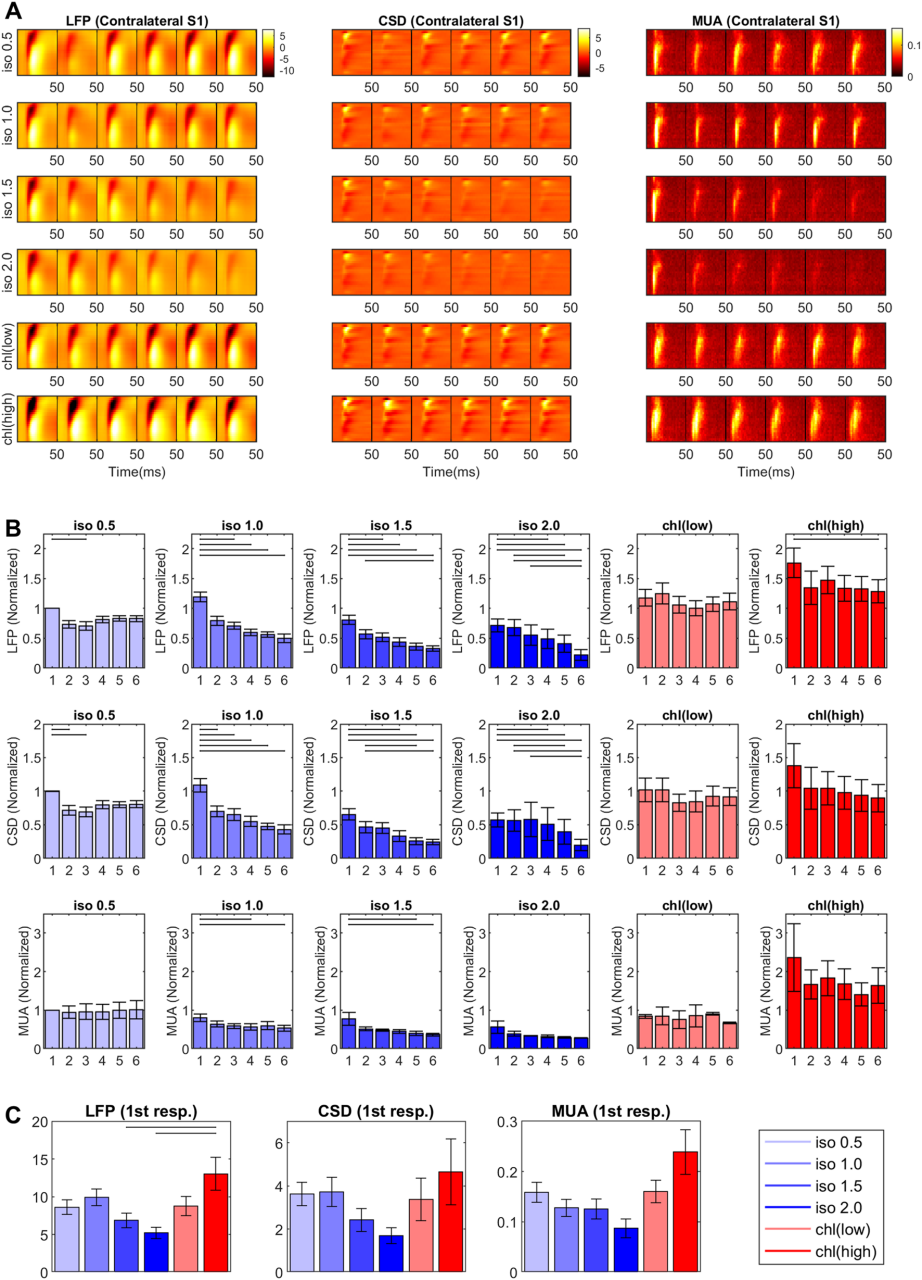
Neural habituation in sensory-evoked responses in a train of forepaw stimulations (1.0 mA, 0.3 μs duration, 3 Hz for 2 s). (**A**) Sequential evoked responses in a stimuli-train (from a representative animal). (**B**) Comparison of the peak amplitude of 6 evoked responses in a train. Response amplitudes in individual animals were normalized to the first (onset) evoked response under isoflurane 0.5% anesthesia. (**C**) Peak amplitude of the first evoked response in a train compared across anesthetic conditions. Horizontal bar: p < 0.05 in post-hoc Tukey’s test. (n = 16; 8 rats × 2 hemispheres).

Anesthesia-affected neuronal habituation of typical evoked responses to repeated stimuli are displayed in Fig. 6A,B. The habituation behavior was pronounced for the isoflurane anesthesia > 1.0% especially when compared to the onset (or 1^st^ in the response train) response. However, such habituation of evoked responses was less evident in the α-chloralose or 0.5% isoflurane anesthesia. The adaptation rate (defined as a ratio between the amplitudes of the first and average of all other responses in the stimulus-train) was not significantly different across 0.5% isoflurane and α-chloralose conditions: 0.76 ± 0.14, 0.93 ± 0.14 and 0.72 ± 0.14 for 0.5% isoflurane, 30 and 60 mg/kg∙hr α-chloralose, respectively (Mean ± S.D. Friedman test). However, higher doses of isoflurane resulted in a profound adaptation of the evoked responses. For high dose isoflurane (> 1.0%), the average response amplitudes dropped to less than 50% of the onset response amplitude: adaptation rate = 0.49 ± 0.13, 0.48 ± 0.17, 0.41 ± 0.16 for isoflurane 1.0%, 1.5% and 2.0%, respectively (paired t-test; p < 0.01 vs. isoflurane 0.5% for all doses).

As shown in Fig. 6C, the onset LFP amplitude of contra-stimulus response significantly dropped to 7.00 ± 3.46 mV and 5.32 ± 2.66 mV at 1.5% and 2.0% isoflurane, respectively, when compared to 8.73 ± 3.67 mV at 0.5% isoflurane (t-test; p < 0.05 for both). On the contrary, statistically significant enhancements of the onset response amplitude was observed for the high dose of α-chloralose (60 mg/kg∙hr) compared to those found in isoflurane 1.5% and 2.0% (Fig. 6C). On the other hand, alteration of the response delay (i.e., time between stimulus and response onset) was not evident except for the contra-stimulus response under α-chloralose 60 mg/kg∙hr, in which the onset delay was significantly longer than 0.5% isoflurane (see Fig. S4B). Moreover, the time delay between contra- and ipsi-stimulus response onsets (i.e., 6 ~ 8 ms) was not significantly affected by either anesthetic agent.

Gathered, isoflurane and α-chloralose induced different cortical excitability to the sensory stimulation. Isoflurane decreased the sensory-evoked response amplitude in a dose-dependent manner, which was characterized with rapid habituation to the repeated stimuli as well as the decreases in the onset response. In contrast, compared to the minimal dose of isoflurane condition (0.5%), α-chloralose did not diminish but rather intensified the evoked responses; particularly notable is the highly enhanced sensory-evoked response in the ipsi-stimulus S1fl. Unlike isoflurane, α-chloralose (particularly in low dose) did not result in rapid habituation of the evoked responses.

## Discussion

### Effects of anesthetics on sensory-evoked activity

The present study was designed to explore possible electrophysiological links between the sensory-evoked response and spontaneous activity in a popular rat model by perturbing the brain using two commonly used anesthetics, isoflurane and α-chloralose. Although a few past BOLD fMRI studies^23,28,30,31^ demonstrated anesthetic modulation of the resting state functional connectivity, its functional relevance to the sensory-evoked activity has not been directly assessed. Related electrophysiological recording studies^28,31^ were usually limited to the evaluation of anesthetic depths (e.g. burst-suppression anesthesia under high dose of isoflurane), lacking the comprehensive assessment of functional connectivity. For addressing this issue, a multi-loci electrophysiological study^18^ by Sellers et al. demonstrated low-dose anesthetic modulation of both resting state functional connectivity and sensory-evoked response in the prefrontal cortex (PFC) and primary visual cortex (V1) in ferret brains. However, the functional roles of such brain connectivity are rather complicated (e.g. involving multi-sensory coupling between visual and auditory information), presumably difficult to relate the obtained connectivity with changes in the sensory processing between these regions. Therefore, the present study examined the bilateral S1fl of rats, the most commonly investigated neural system in animal fMRI and functional connectivity studies. As stated above, despite the frequent use of isoflurane or α-chloralose in such studies, effects of these two anesthetics have not been either systemically investigated or compared. In this regard, the experiments were specifically focused to fill this gap and elucidate the effects of isoflurane and α-chloralose on both the spontaneous neural activity and sensory-evoked responses for a wide range of anesthesia depths.

Although some inhibitive mechanisms are known to be shared by both anesthetics at the molecular and receptor levels (e.g., GABA_A_)^36,43^, a clear understanding of the neurophysiological alterations is still a work in progress, and the effects of anesthesia on the complex brain activities remained to be unveiled. In this work, two-site electrophysiological recordings at the bilateral cortices were acquired using a pair of laminar electrodes to gather signals throughout the whole cortical thickness to quantify the anesthetic effects on the well-known sensory evoked response and resting state FC within and between these regions.

In brief, both anesthetics largely affected the electrophysiological activities of the evoked responses to electrical forelimb stimuli. Interestingly, isoflurane decreased the evoked S1fl response amplitude in a dose-dependent manner, whereas α-chloralose significantly increased the evoked response in comparison to that acquired under isoflurane anesthesia. The spontaneous neuroelectric activities and resultant FC were also differently affected by either anesthetic. Such changes under anesthesia raise a challenge whether the variable evoked-response and/or FC strengths under anesthesia (or altered neurophysiology) provide genuine understanding and representation of the intrinsic behavior and/or integrity of neuronal networks.

On a related note, the evoked response magnitude under light anesthesia has been reported to be dependent on the appearance of periodic UP/DOWN states of neurons, typically induced by light anesthesia^38,39^. However, upon quantification of the evoked responses in the present study, synchronized analysis of the evoked responses based on the identification of UP/DOWN states was not performed since the dose range of GABAergic anesthetics is known to be very narrow between stable sedation for eliciting UP/DOWN states and emergence of burst-suppression activities^29,44^. The anesthesia levels adopted in this study were not optimized for this purpose, in which resting state electrophysiology for either anesthetic did not exhibit known characteristics of consistent periods of UP/DOWN states (as shown in spectrograms in Fig. S6). Therefore, the synchronized analysis^45,46^ was not performed in the present study.

Upon comparing two anesthetics in other previous reports, α-chloralose has been preferred over isoflurane in the animal fMRI studies of sensory-evoked responses, due to the fact that α-chloralose delivers well-defined, consistent fMRI responses with a reliable SNR. In contrast, isoflurane often fails to produce robust activations or requires much stronger stimulation^47,48^. Although the isoflurane results are similarly comparable between fMRI and electrophysiology, the matching conclusion of these observations from electrophysiology and fMRI is probably confounded with a potent vasodilation effect of isoflurane, which disrupts the normal neurovascular coupling and affects the sensory-evoked fMRI responses^49^. Such possibility requires the elucidation of underlying electrophysiological events to avoid misinterpretation of the results from other indirect measures of neuronal events. On the other hand, previous resting state fMRI and electrophysiology studies also demonstrated a distinctive difference between two anesthetics in the construction of resting state FC. Both fMRI and electrophysiological studies similarly reported that non-specific correlation pattern induced by isoflurane while α-chloralose was shown to preserve the connectivity between the established brain areas^30,33^. Although such differences have been documented, anesthetic effects on the spontaneous FC was not yet comprehensively investigated in relation to the evoked response characteristics. The current study examined this gap by providing the quantitative description of the alteration in electrophysiology.

### Effects of anesthesia on spontaneous brain activity and functional connectivity

As shown in Fig. 4, the band coherence analysis revealed that spontaneous oscillations in the slow wave, delta and theta bands, are main contributors in the formation of interhemispheric correlation across the tested anesthesia states. In general, the observation agrees with the general acceptance that the low frequency oscillations are underlying mediators of the FC in the resting state brain^24,50^. Contrary to the diverging effects of isoflurane (i.e., decrease) and α-chloralose (increase) on the evoked response (Fig. 5), higher doses of isoflurane (1.0–2.0%) and α-chloralose elevated the spectral power when compared to 0.5% isoflurane results. The overall spontaneous activity tended to increase by both anesthetics across delta ~ beta frequency bands (Fig. 2B). Spatially, greater enhancements concentrated in relatively deep layers (i.e., the granular and infragranular layers). As neurons in these deep layers are known to be heavily associated with the signal reception and transmission to adjacent cortical columns and/or extracortical structures, such heightened spontaneous activities under anesthesia might result in different interpretations of the FC among the affected brain areas^51^. Indeed, the anesthesia-induced changes in spontaneous activity ultimately resulted in the anesthetic type- and dose-dependent variations of interhemispheric correlation and spatial pattern of it (Fig. 3).

For isoflurane, previous studies demonstrated that the altered interhemispheric correlation was characterized by non-specific, global synchronization of slow oscillations with the burst suppression of neuronal activities as a source mechanism (Fig. 1 and Table 1)^28,33^. Similarly in the current study, such global synchronization increasingly manifested as the isoflurane dose increased and resulted in high cross-correlation across the entire cortical thickness (Fig. 3). As the isoflurane dose was increased (1.0%), both the spontaneous neural activity and suppression progressively increased further, in which the increases were especially concentrated in the mid and deep layers (Fig. 2). At the same time, the interhemispheric correlation gradually became nonspecific across the entire cortical layers. However, more stringent z-statistics revealed presence of the layer-dependent interhemispheric correlation pattern even in high dose conditions, the extent and strength of which increased as the dose increased (Fig. 3C,E,F). Such presence of layer-specific correlation patterns (especially in deep layers) suggested that the spontaneous activity arises from the distinct neural circuitry linked with fine laminar arrangement between homotopic regions. Accordingly, the spontaneous activity along the cortical depth is transmitted via corpus callosum, the functions of which are little-affected by isoflurane anesthesia^32^. Given these findings, we posit that the burst suppression under isoflurane-induced anesthesia non-selectively masks out the existing intrinsic activity without interrupting the actual FC. Nonetheless, as shown in the z-statistics, the layer-dependent correlation likely strengthened as the isoflurane dose increased, unlike the evoked responses.

On the other hand, α-chloralose maintained the overall resting state FC compared to the 0.5% isoflurane baseline (Figs. 3 and 4). As shown in Fig. 2, the band power increases caused by α-chloralose in spontaneous oscillations occurred mostly in delta, theta and alpha bands. However, significant increases in these band powers (e.g., theta and alpha) did not translate into the increased coherence in corresponding frequency bands, only exhibiting the layer-dependent correlation pattern similar to that measured in 0.5% isoflurane (Fig. 4A). Therefore, the power changes in these frequency bands by α-chloralose are probably local.

In addition, the gamma band activities were not affected by either anesthetic. As the gamma band power has been implicated for the long-range FC including interhemispheric correlation in primate and human studies^26,52,53^, the observed robustness of gamma band coherence in relation to the beta coherence might project a similar role of gamma band oscillations in the construction of interhemispheric FC of rodent brains.

Gathered from these observations, we may further hypothesize the power change in the band activity by anesthetics does not necessarily affect the strength and spatial profiles of the interhemispheric FC, independent of the evoked response characteristics. Moreover, although not well understood, the current data suggests that inhibitory actions on neurons and interneurons are likely independent between the circuits associated with either sensory evoked processing or resting state activity, which are differently affected by the choice of anesthetics^32,54^.

### Habituation of responses to repeated stimuli

During the forelimb stimulation, we observed that isoflurane reduced the average contra-stimulus response (i.e., activation in the hemisphere contralateral to the stimulated forelimb) in all measures (i.e. LFP, CSD, MUA). Such decreases in the activation amplitude were largely due to the rapid habituation of the responses over repeated sensory stimuli whereas a single stimulus after sufficient recovery time resulted in the activation much less affected by the increase of the isoflurane dose (See Fig. 6C). Similarly, other studies also reported decreased evoked response or rapid adaptation to repeated sensory stimuli under isoflurane anesthesia in rodent models^55^ as well as in humans^56^. On the other hand, the evoked activation during 0.5% isoflurane and anesthesia by α-chloralose were characterized by robust, non-habituating responses.

In addition, α-chloralose significantly increased the levels of response amplitudes compared to those observed in isoflurane-induced anesthesia. Intuitively, anesthesia states are thought to produce a reduction of CNS responsiveness to external stimuli, mainly via activation of inhibitory GABA_A_ receptors^29,35^. However, apparent elevation of the response activity by α-chloralose in the current study indicates unusual changes in neurophysiology and/or the signal processing mechanism. In fact, such dichotomous results in habituation between isoflurane and α-chloralose suggest that a choice of anesthesia could differently disrupt the intrinsic sensory processing of cortical neurons. These changes are likely related to the modulation of the neuronal excitability and disparate effects of anesthetics on the neurochemical cascades during post-activation refractory period including the changes in the action potential threshold^57,58^. In this regard, other previous studies suggested that the increase or decrease of cortical excitability might be caused by excitatory/inhibitory imbalance in the neuro-signal circuitry in association with neuropsychiatric diseases^59,60^. The altered habituation or elevation of evoked activities by anesthesia in response to repeated sensory stimuli might be important to address neurological changes, warranting further investigations in evaluation of the involved mechanisms and possible damage and/or benefits.

### Contra- vs. ipsi-stimulus responses to stimulation

Although much smaller in amplitude compared to the contra-stimulus response counterpart, the ipsi-stimulus responses were consistently detected and always accompanied the contra-stimulus activation across all anesthetic conditions. In our previous electrophysiological study^32^, we reported that such ipsi-stimulus responses in S1fl were detectable and delayed by 6 ~ 8 ms to the contra-stimulus response, suggesting that the signal transmission from contra-stimulus activation occurred via the corpus callosum and resulted in the time-delayed interhemispheric activation^32,61^. Similarly, such ipsi-stimulus responses have been observed in both BOLD fMRI and EEG studies for primates^62^. In human subjects, the neuroelectric ipsi-stimulus evoked activity (e.g., EEG) was found to be delayed by ~ 15 ms in the homologous area in the contralateral hemisphere^63^.

In the current study, for isoflurane, amplitudes of such transcallosal ipsi-stimulus responses (i.e., LFP and CSD; see Fig. 5) progressively decreased as the dose of isoflurane increased, which was generally proportional to the concomitant decrease in the contra-stimulus responses. On the contrary, both contra- and ipsi-stimulus responses under α-chloralose appeared to increase as the dose increased and were substantially greater than those observed under isoflurane anesthesia. More noteworthy, the increases in the ipsi-stimulus response by α-chloralose were not proportional to and largely exceeded the percent increases of the contra-stimulus responses unlike those observed under isoflurane. Additionally, regardless of the anesthetic choice and dose, the interhemispheric delay was not affected by the level of consciousness. It indicates that the axonal conductance was little varied by the choice and dose of anesthetics, thus revealing that the effective connectivity was not affected by the efficiency of signal transmission. The pronounced, disproportional increase of ipsi-stimulus activation by α-chloralose is likely local in nature and derived from the aforementioned influences on the cortical excitability and excitatory/inhibitory imbalance.

One interesting note is that, unlike primate studies, the ipsi-stimulus fMRI activation was not observed under the same experimental setup (i.e., α-chloralose and electrical forelimb stimulation) in previous studies^64^. Despite the disproportionately amplified electrophysiological activity by α-chloralose anesthesia, the ipsi-stimulus fMRI activation has not been observed even using the contrast agent-enhanced CBV-weighted fMRI, which typically provides an activation SNR much greater than that found in BOLD fMRI^65^. In this regard, we suggest that such ipsilateral electrophysiological responses in rodents may not accompany typical neurovascular activation cascades. Combined with the lack of corresponding fMRI signals, the observed changes in ipsi-stimulus neuroelectric activation could be important upon the interpretation of effective connectivity in neuroimaging studies. We believe that future studies are warranted for investigating significance and proper application of such activities in the FC analysis^66^.

### Possible mechanisms for altered evoked response

Previously, the study by Detsch et al.^67^ showed that spiking activity of thalamo-cortical relay neurons in response to the repetitive somatosensory stimulation was decreased by isoflurane in a dose-dependent manner. They also showed that the sustained responses during the stimulus train largely diminished over the repeated stimuli while the onset response in the stimulus train was preserved. Similarly in the present study, although reduced with higher doses of isoflurane, almost no statistical differences were found among the onset responses (Fig. 6C). Therefore, one can posit that an insufficient refractory period, and thus, the compromised polarization by isoflurane action likely is a possible cause for the habituation of the repetitive neuronal responses. As regards to mechanism involving networks, isoflurane as a GABA_A_ agonist is suggested to interact with the thalamic inhibitory network, in which the GABAergic thalamic reticular neurons may be intercalated into the reciprocal thalamo-cortical connections via collaterals^67^. Additionally, short-term depression at the thalamo-cortical synapses can also be implicated as a neuronal mechanism for rapid adaptation of the cortical sensory responses^68^. On the other hand, in the current study, anesthesia by low dose isoflurane at 0.5% or α-chloralose, also known to be a GABAergic anesthetic, exhibited little habituation to repetitive stimuli. The absence of suppression period and possible stability of the refractory period could be related with the current findings of weakened habituation under a-chloralose and 0.5% isoflurane anesthesia. In line with such observations, a significant increase of the onset response magnitude by high dose α-chloralose (compared to 0.5% isoflurane) may lead to a hypothesis that the frequency and duration of hyperpolarized DOWN states (that are intrinsically different from suppression) increase by the α-chloralose anesthesia, thus eliciting considerably large evoked responses. Although speculative, the finding of increased neuronal excitability induced by the altered baseline neurophysiology (i.e. DOWN state) is further advocated by the increased contra-stimulus and substantially large ipsi-stimulus responses.

## Limitations

Following limitations should be considered in the present study. First, the order of anesthetic conditions was partially fixed. In all animals, we examined (i) the varying doses of isoflurane first and then switched to (ii) the α-chloralose. Isoflurane is a volatile anesthetic agent, and its anesthetic effect is rapidly reversible. Thus, we were able to randomize the order of isoflurane conditions (0.5%, 1.0%, 1.5% and 2.0%). However, α-chloralose is intravenously infused and requires a long switching period (45 min) to achieve a stable level of anesthesia. To optimize the duration of experiment, α-chloralose conditions were always investigated after the isoflurane study and from the low (30 mg/kg∙h) to the high dose (30 mg/kg∙h) to avoid any residual influence after the high dose of α-chloralose. The prolonged experiment in latter anesthetic conditions might affect the electrophysiological recording.

Second, direct comparison with the awake condition was not feasible in the present study due to apparent limitations in our equipment setting (i.e., movement). Nonetheless, we compared measurements across varying anesthetic conditions including the light sedation with 0.5% isoflurane, with intention to expand our understanding of the different anesthetic effects (isoflurane and α-chloralose) and describe spontaneous activity and sensory-evoked responses under these conditions. We often used 0.5% isoflurane (the minimal dose condition) as a baseline for comparison in some cases (e.g. Fig. 3C). However, we must acknowledge that 0.5% isoflurane is not ideal as the awake state. Characterization of the awake condition still remains unfulfilled for future investigation and will be very helpful in the more complete examination of anesthetic effects.

Lastly, there were a large number of multiple comparisons when considering all variables such as anesthetic conditions, cortical depths and frequency bands altogether. We utilized omnibus Friedman tests to prevent type I error from multiple comparisons related with a single study variables (e.g. comparing across anesthetic conditions). However, a multiple comparison issue may still emerge for some cases of multiple statistical tests in the present study such as running statistical tests about anesthetic conditions for 3 different cortical layers simultaneously.

## Conclusion

The present rodent electrophysiology study quantitatively assessed the effects of anesthesia by two popular anesthetics on the (i) evoked response and (ii) resting state activities. The anesthetic modulations revealed that both resting state FC and evoked response were nonlinearly altered, in which anesthesia-induced changes in the neuronal excitability did not exert influences on the strength of FC. The findings strongly encourage that the evaluation of baseline neural state and understanding of underlying neurophysiology should precede the determination of FC and sensory information processing efficiency.

## Methods

### Animal preparation

The animal study information was provided in accordance with recommendations in ARRIVE guideline^69^. All experimental procedures were approved by the Institutional Animal Care and Use Committee (IACUC) in Seoul Asan Medical Center. All experiments were performed in relevant national guidelines and regulations as well. A single group of animals were compared across varying anesthetic conditions (see below for details). Normal, healthy adult male Sprague–Dawley rats (n = 8, 250–350 g) were used for the electrophysiology experiment. The animals were housed in a group (2 ~ 4 animals per cage) in the conventional housing condition.

The animal was trained for the head restraint before the experiment as we utilized low dose of anesthetic agent (e.g. 0.5% isoflurane in medical air) in the early periods in the electrophysiological recording experiment. We utilized the head-restraining system as described in Fa et al.^70^. In the initial surgery using isoflurane 1.5%, we placed the stainless steel nuts (one in the frontal area and two in the occipital area) using stereotaxic frame and secured them to the bone surface using dental cement. The restraining bridge was attached to the nuts in later training and experiment. The areas of skull overlying the bilateral primary somatosensory cortices (S1) were exposed and thinned as well. The experiment was performed after 1 ~ 2 weeks of training with the head-restraining system; the rat was suspended as described in Fa et al.^70^ while its head was immobilized in the stereotaxic frame using the restraining platform. The dose of isoflurane was gradually decreased to 0.5% during the training.

During the surgery, the animal was anesthetized using isoflurane 1.5% in medical air. The areas of skull and dura matter overlying the bilateral S1 were removed for the electrophysiological recording. Lidocaine of 2 mg was applied on the areas for reducing possible pain and discomfort. The lateral tail vein of the animal was cannulated for later administration of intravenous anesthetic agent (i.e. α-chloralose). The body temperature (37.0 °C) was maintained with a temperature-controlled heating pad placed under the rat’s torso. The respiration rate and the body temperature were monitored and carefully maintained throughout the experiment. The duration of surgical preparation before the electrophysiological recording was less than 1 h.

### Electrophysiological recordings

We performed the electrophysiological recordings in the forelimb region of the bilateral primary somatosensory cortices (S1fl) using a pair of linear multi-electrode arrays. The multi-electrode array (Plexon Inc., U-probe, 24 channels with 25 μm electrode diameter) has 23 contact points with a 100 μm spacing between each contact, which covered the entire depth of the cortex^71^. Impedance of the electrode was 54 kΩ (standard deviaion of 11 kΩ). Each electrode array has separate contacts for ground and reference channels. The reference electrode was placed on the posterior end of the skull, and the ground channel was connected to the metal stereotaxic frame. The electrode was intruded at the S1fl region (4.0 mm lateral and 0.5 mm anterior from the bregma) with the angle of 5 degrees to place the electrode perpendicular to the cortical layers. The depth of electrode was identified with (1) the polarity of spontaneous neural activity at the cortical surface and (2) the current sink in the sensory-evoked response in a similar method with Maier et al.^72^ During the typical recording of spontaneous LFP activity, a reversal of polarity and small LFP fluctuations were consistently observed exactly at the cortical surface, which was visually marked as reference for the electrode depth. Following the analysis of CSD, the depth of electrode was further confirmed by observing two current sinks in the sensory-evoked responses as exhibited in the CSD maps of Fig. S7. Based on these demarcations, the cortical layers were aligned and divided into the following three layers: (1) supragranular layer (layer 1 ~ 3): 0 ~ 500 μm, (2) granular layer (layer 4): 600 ~ 900 μm, (3) infragranular layer (layer 5 ~ 6): 1000 μm or deeper^73,74^.

The extracellular LFP signals were amplified and filtered between 0.1 and 500 Hz using a custom-built amplifier equipped with analog butterworth filter circuits. The LFP was recorded with a sampling rate of 1,000 Hz. The MUA data was simultaneously recorded as well with a sampling rate of 5,000 Hz with the custom-built amplifier (amplification rate of 10,000 with a built-in butterworth bandpass filter circuit).

For each anesthetic state, we recorded electrophysiological activity in the following conditions: (1) 5 min during rest and (2) 2 min during forelimb stimulation. The electrical forepaw stimulation was administered through a pair of 30-gauge syringe needles intruded at animal’s forepaw. The magnitude, duration and frequency of the stimulation was controlled by Grass stimulator with the isoelctric stimulation module (~ 1.0 mA, 3 Hz, 12 pulses per train, duration of each pulse of 0.3 ms, inter-train interval of 6 s).

### Anesthetic modulation

The anesthetic condition was systematically modulated. Lidocaine was applied around the opening of the skull to prevent pain and discomfort of the animal in light sedation (< 1.5% isoflurane). At the beginning of the experiment, isoflurane was gradually lowered to 0.5% for more than 30 min. Then, the dose of isoflurane was modulated across 0.5%, 1.0%, 1.5% and 2.0% in a randomized order with an interval of 15 min between different dose conditions. Recording of evoked response and resting state activity in each anesthetic condition was conducted after this acclimation period (15 min), which was much longer than the recovery time from the isoflurane anesthesia in rats (33 ~ 60 s for isoflurane 1.5%, up to 6 min for isoflurane 2 ~ 3%)^75,76^. Before switching the anesthetic to α-chloralose, isoflurane dose was lowered to 1.0%, and the recovery of neural activity was confirmed (i.e. diminishing of burst-suppression). Then, anesthesia was switched to continuous intravenous infusion of α-chloralose, in which α-chloralose dose was initially set at 30 mg/kg∙hr and then increased to 60 mg/kg∙hr later. Electrophysiological recording was conducted after the acclimation period of more than 45 min for each α-chloralose anesthesia condition, which was close to a 50-min interval in the previous study using intravenous bolus injections^24^.

### Data analysis

All data analysis for the LFP and MUA signal was conducted using custom-written MATLAB code (The Mathworks; Natick, MA). The offline digital bandpass filter (FFT and inverse FFT for 0.1 ~ 100 Hz) between 0.1 and 100 Hz was applied on LFP signals to remove low-frequency drifts and other noises. We also utilized a band-stop filter between 59 and 61 Hz to reject 60 Hz artifact. Bad channels of recording with large drift (> 20 mV) were identified and corrected by interpolation of the signals from adjacent channels. The evoked responses were averaged at the onset of forelimb stimulation to exclude spontaneous background activity. Furthermore, the response magnitude to each stimulus in a train of stimuli was averaged and compared against each other (see Fig. 6) by averaging 12 trains for 2 min of sensory-evoked response recording in each condition.

The MUA recordings were first filtered between 300 ~ 2,500 Hz, which were rectified and smoothed with a time window of 10 ms so that it reflects the power of high-frequency spike activity during this window. The sensory-evoked MUA response was calculated by averaging the estimated MUA activity during the electrical forepaw stimulation (0 ~ 200 ms). Then, the peak of the average MUA response within a time window of 0 ~ 50 ms was used to compare the amplitude of the evoked MUA responses. On the other hand, the spontaneous MUA activity was not either robust or clearly distinguishable as we were not able to enhance the SNR by averaging across epochs. In our experiment settings, the sensory-evoked responses were robustly observable in each epoch, but the baseline MUA activity did not show spontaneous fluctuation in a comparable amplitude. Thus, we excluded spontaneous MUA activity from further analysis.

The current source density (CSD) was estimated by the second derivatives of the LFP signal along the cortical depth, as described in the previous literature by Chapman et al.^77^ The CSD (in arbitrary unit) was numerically computed as follows:

$$C(z)=-\sigma \frac{V\left(z-\delta \right)-2V\left(z\right)+V\left(z+\delta \right)]}{{\delta }^{2}}$$

The spectral power of the resting state LFP signal at each channel was calculated by the Fast Fourier Transform algorithm in MATLAB for 5 min of resting state activity in each anesthetic condition. We also calculated spectral power at the following frequency bands: slow wave (0.1 ~ 1 Hz), delta (1–4 Hz), theta (4–8 Hz), alpha (8–13 Hz), beta (13–30 Hz) and gamma (30–100 Hz).

Zero-lag cross correlation was calculated between each channel of the resting state recording (within and between bilateral cortices). Magnitude-squared coherence in specific frequency bands (delta to gamma bands as described above) was also estimated using Welsch’s method (window length of 10 s, number of windows > 25).

The suppression period was quantified as the description of burst-suppression activity. The burst period was defined as the total recording period minus the suppression period, which was used for the analysis of burst activity^78^. Specifically, suppression periods were defined as periods with LFP amplitudes less than 0.1 mV/ms for a period of 500 ms or longer. If there is another LFP change (> 0.1 mV for 1 ms) within 500 ms, such short isoelectric periods were merged into an active period. In anesthesia with isoflurane 0.5% or α -chloralose, such isoelectric periods were always shorter than 500 ms, thus total recording period was identified as a single active state. Burst suppression ratio was calculated as the total duration of suppression periods divided by the total length of the LFP recording. Duration of suppression periods was estimated as the duration of individual suppression (isoelectric) periods which was defined as above.

For quantitative comparison, average values of the interhemispheric correlation coefficients between the spontaneous LFP activities at the same depth of the bilateral S1fl’s (e.g., averaged correlation values from 23 pairs of LFP channels spanning 0 ~ 2200 μm depth in the bilateral cortices) were calculated. We also estimated the average correlation coefficient recorded at different depths of the bilateral S1fl’s (depth difference ≥ 100 μm), which reflected the interhemispheric correlation nonspecific to the cortical depth. Then, the ratio between the former (layer-to-layer correlation) and latter values (nonspecific to the cortical depth) was calculated for describing the layer-specific distribution in interhemispheric correlation values as shown Fig. 3G. Similarly, the coherence and associated layer-specificity were also analyzed per frequency band (see Fig. 4).

Friedman test, a nonparametric test alternative for a repeated measure ANOVA, was conducted to compare characteristics of spontaneous activity and sensory-evoked response along the different anesthetic conditions. Tukey’s test was used for post-hoc analysis. The peak amplitude in the sensory-evoked response (Fig. 5B) was estimated in the time window of 0 ~ 50 ms after the forepaw stimulation. The highest peak amplitudes along the cortical depth were compared in the post-hoc analysis (Fig. 5C). All data analysis was conducted using MATLAB (Mathworks, Natick, MA).

## Supplementary Information

Supplementary Information.
