# Supplementary material for: Anesthetic modulations dissociate neuroelectric characteristics between sensory-evoked and spontaneous activities across bilateral rat somatosensory cortical laminae
Source: Sci Rep. 2022 Jul 8;12:11661. doi: 10.1038/s41598-022-13759-0 (PMC9270342; doi:10.1038/s41598-022-13759-0)

**[Supplementary Information]**

**Anesthetic modulations dissociate neuroelectric characteristics between sensory-evoked and spontaneous activities across bilateral rat somatosensory cortical laminae**

Kwangyeol Baek^1&^, Chae Ri Park^2&^, Siwan Jang^3^, Woo Hyun Shim^2,4*^, Young Ro Kim^5,6*^

1 School of Biomedical Convergence Engineering, Pusan National University, Busan, Republic of Korea

2 Department of Medical Science, Asan Medical Institute of Convergence Science and Technology, Asan Medical Center, University of Ulsan College of Medicine, Seoul, Republic of Korea

3 Washington University in St. Louis, St. Louis, MO

4 Department of Radiology, Asan Medical Center, College of Medicine, University of Ulsan, Ulsan, South Korea

5 Athinoula A. Martinos Center for Biomedical Imaging, Massachusetts General Hospital, Charlestown, MA

6 Department of Radiology, Harvard Medical School, Boston, MA

^&^ K. Baek and C. Park contributed equally in this study.

^*^ Correspondence: Young R. Kim, [spmn@nmr.mgh.harvard.edu](mailto:spmn@nmr.mgh.harvard.edu); Woo Hyun Shim, swh@amc.seoul.kr

**Supplementary Figure 1.** Spectral power analyses show the frequency distribution of the spontaneous LFP activity across layers. As the anesthetic dose increased, the frequency of the peak power decreased for isoflurane (n = 16; 8 rats x 2 hemispheres).


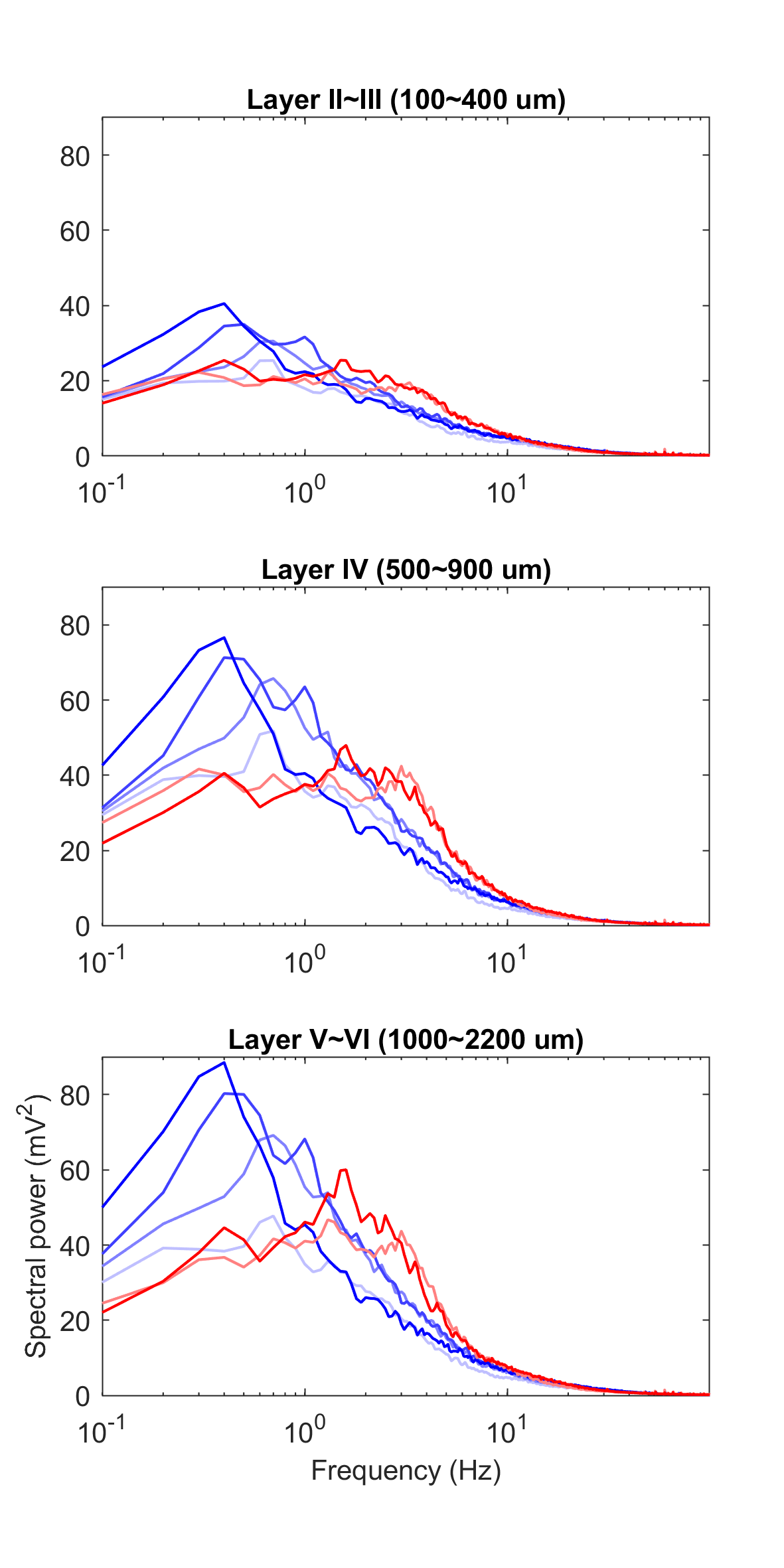


**Supplementary Figure 2.** Laminar correlation in spontaneous activity during the burst periods and the suppression periods in the isoflurane anesthesia (isoflurane 1.0%, 1.5% and 2.0%). The burst periods and the suppression periods were identified for each anesthetic condition in individual animals, then zero-lag correlation was computed from the concatenated time series of the burst periods or the suppression periods, respectively. (A) Pearson’s correlation coefficient *r* across spontaneous LFP activity in bilateral S1fl. (B) Z-statistics calculated by Fisher transformation.


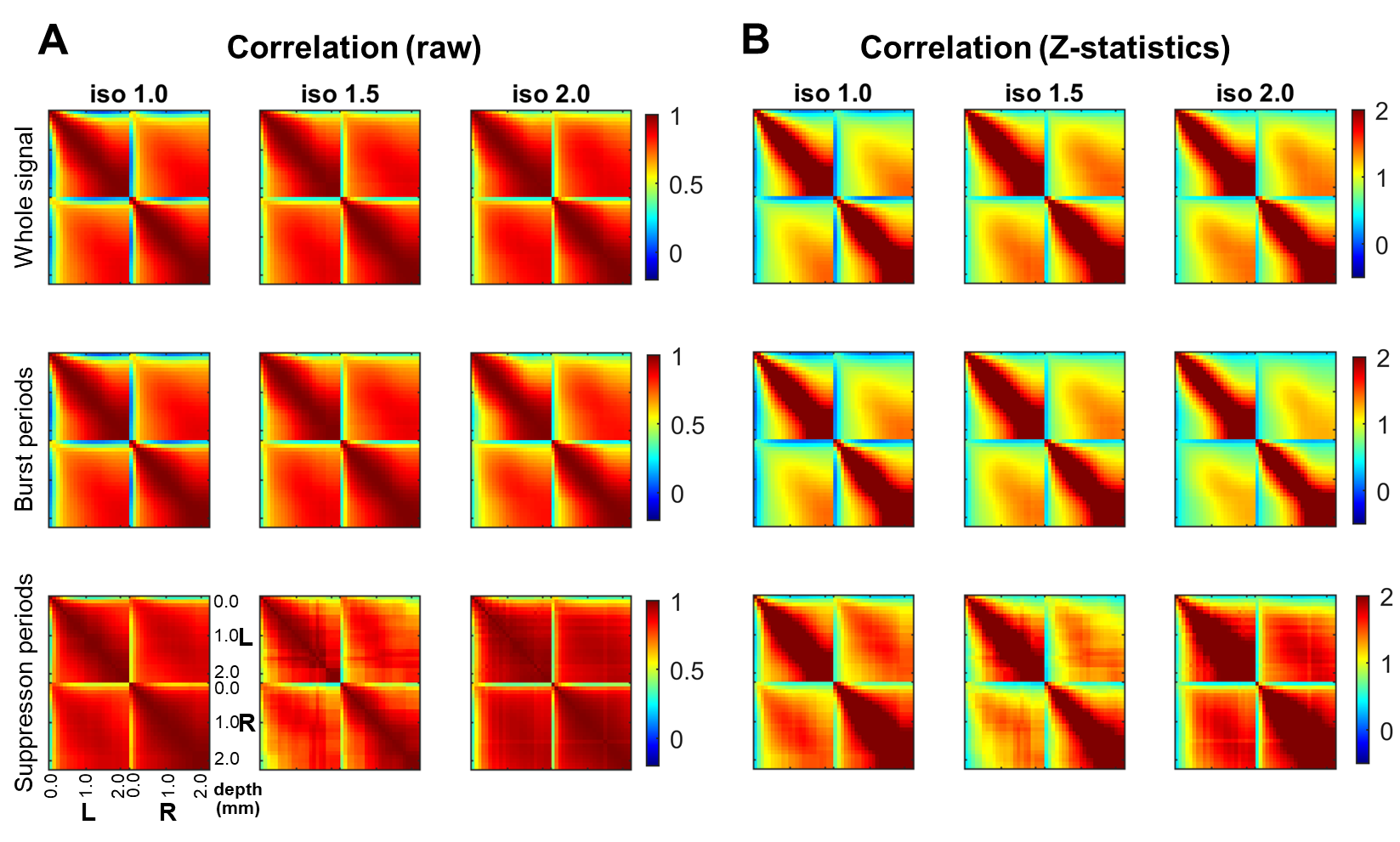


**Supplementary Figure 3.** Correlation coefficients (z-statistics) across cortical layers were compared across varying anesthetic conditions. SG: supragranular layer (depth: 100~500 μm), G: granular layer (depth: 600~900 μm), IG: infragranular layer (depth: 1000 μm or deeper). Average of correlation value (z-statistics) were computed for each pair of cortical depths. Horizontal bar: p < 0.05 in post-hoc Tukey’s test

**
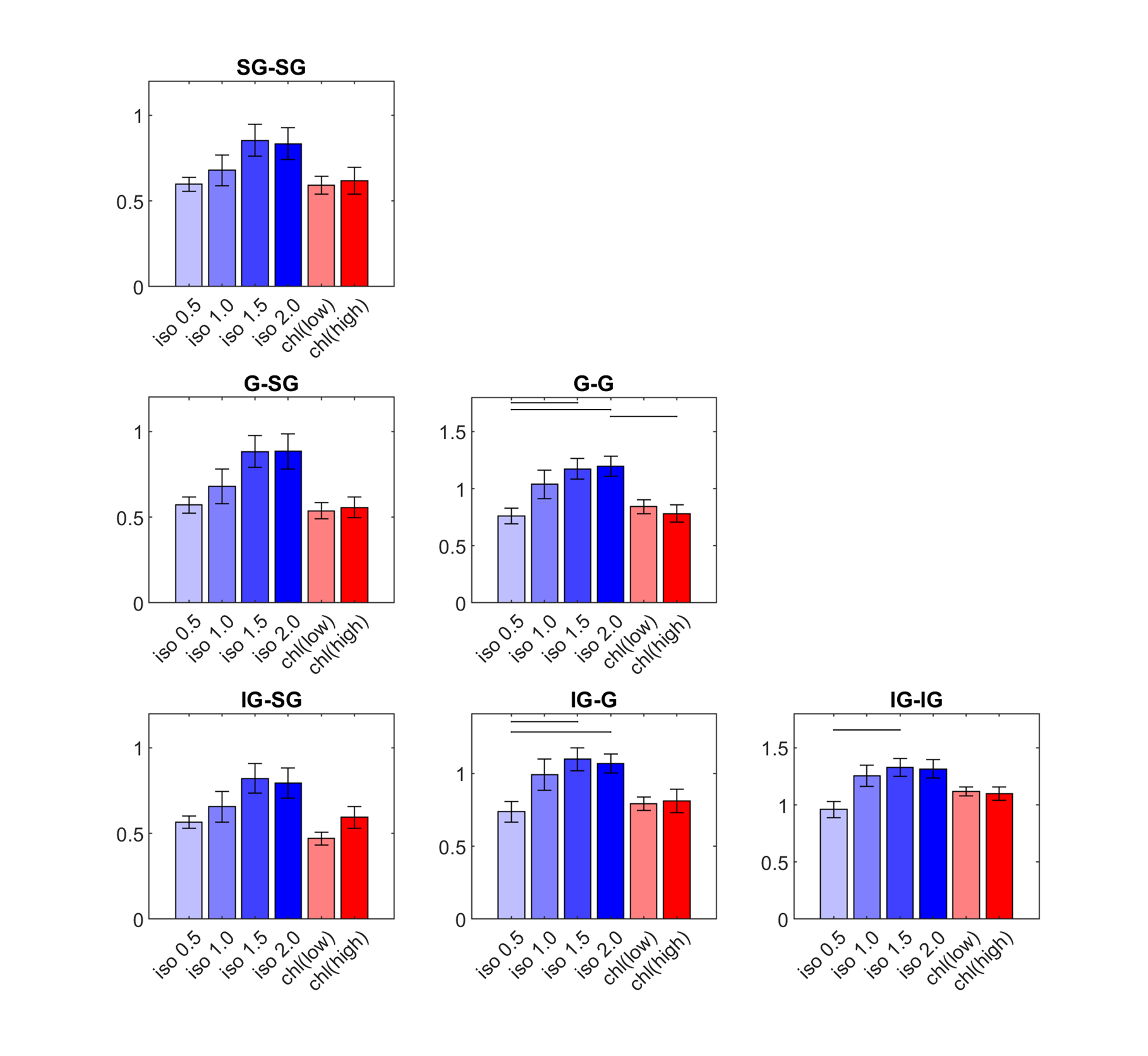
**

**Supplementary Figure 4.** Sensory-evoked responses and interhemispheric delay across varying anesthetic conditions (A representative animal). (A) Traces of evoked response along cortical depth. Ipsilateral response was magnified by 5 folds for better visualization. (B) Response onsets and interhemispheric time delay in evoked responses in the bilateral S1fl areas. Only contra-stimulus response onsets in isoflurane 0.5% vs. a-choloralose 60 mg were significantly different (p < 0.01, post-hoc analysis). n = 16; 8 rats x 2 hemispheres.

**
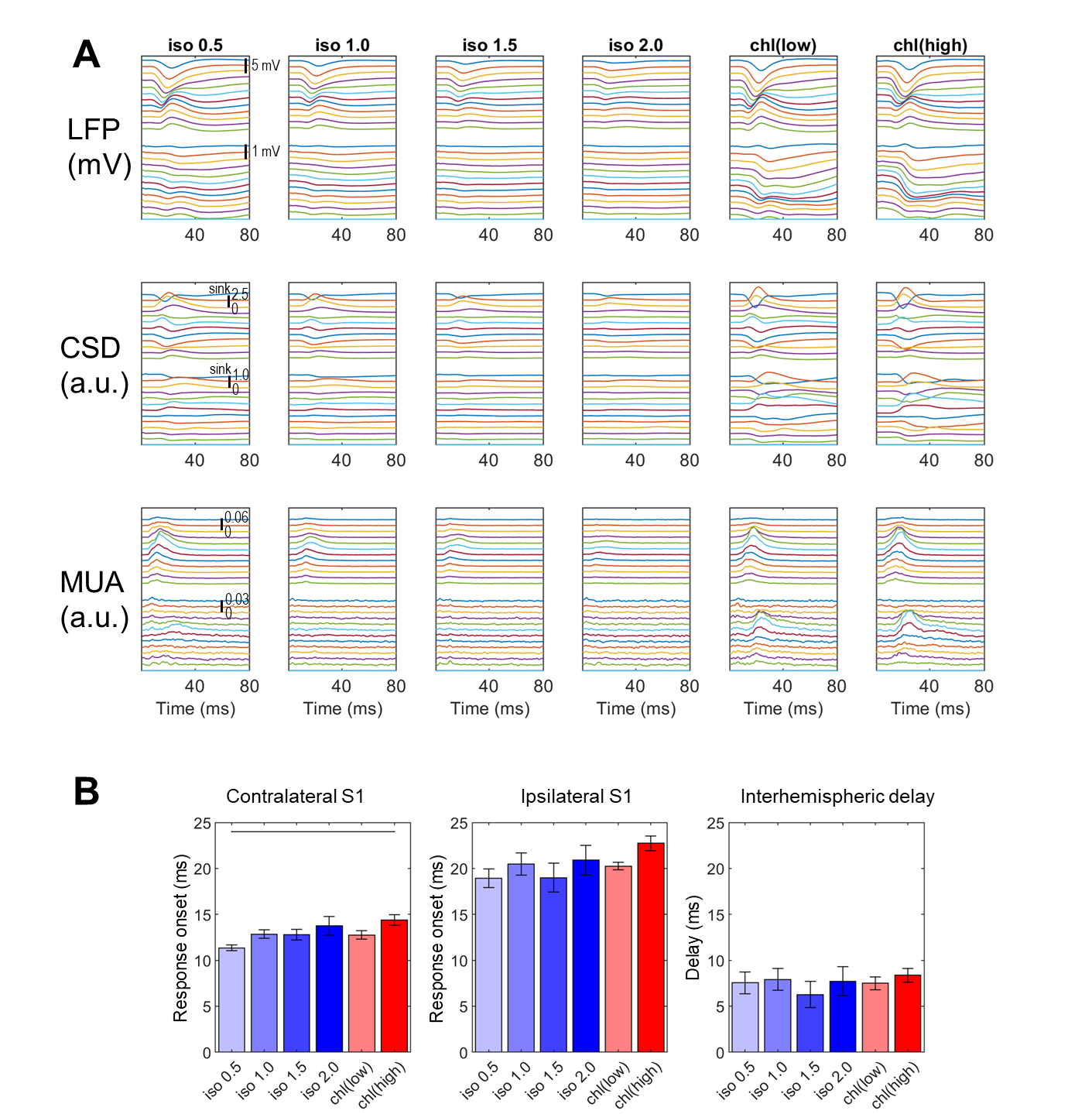
**

**Supplementary Figure 5.** Average evoked response from left forepaw stimulation (Top) and right forepaw stimulation (Bottom). Left and right S1 are reordered to be the same arrangement with Fig.5A. Note that the different colormap scales for contralateral and ipsilateral regions. Both side of S1fl exhibited similar evoked responses robustly.

**
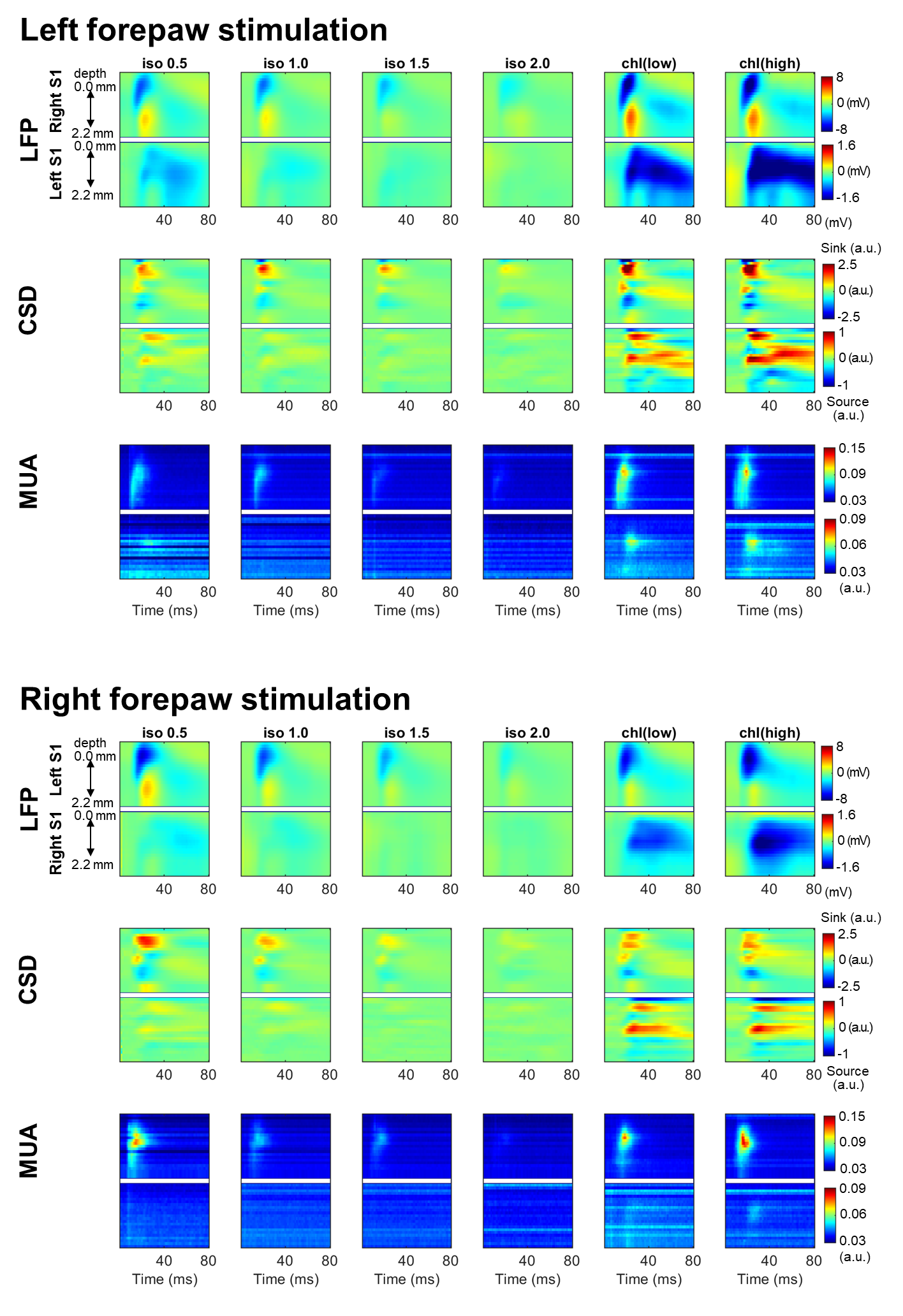
**

**Supplementary Figure 6.** Spectrogram analysis of the spontaneous LFP activity at different depth. (A) Layer 2-3 (depth 100~500 μm). (B) Layer 4 (depth 600~900 μm). (A) Layer 5-6 (depth 1000~2200 μm). Our spontaneous LFP recording data did not show a regular pattern of up/down states. A result from a representative animal.

**
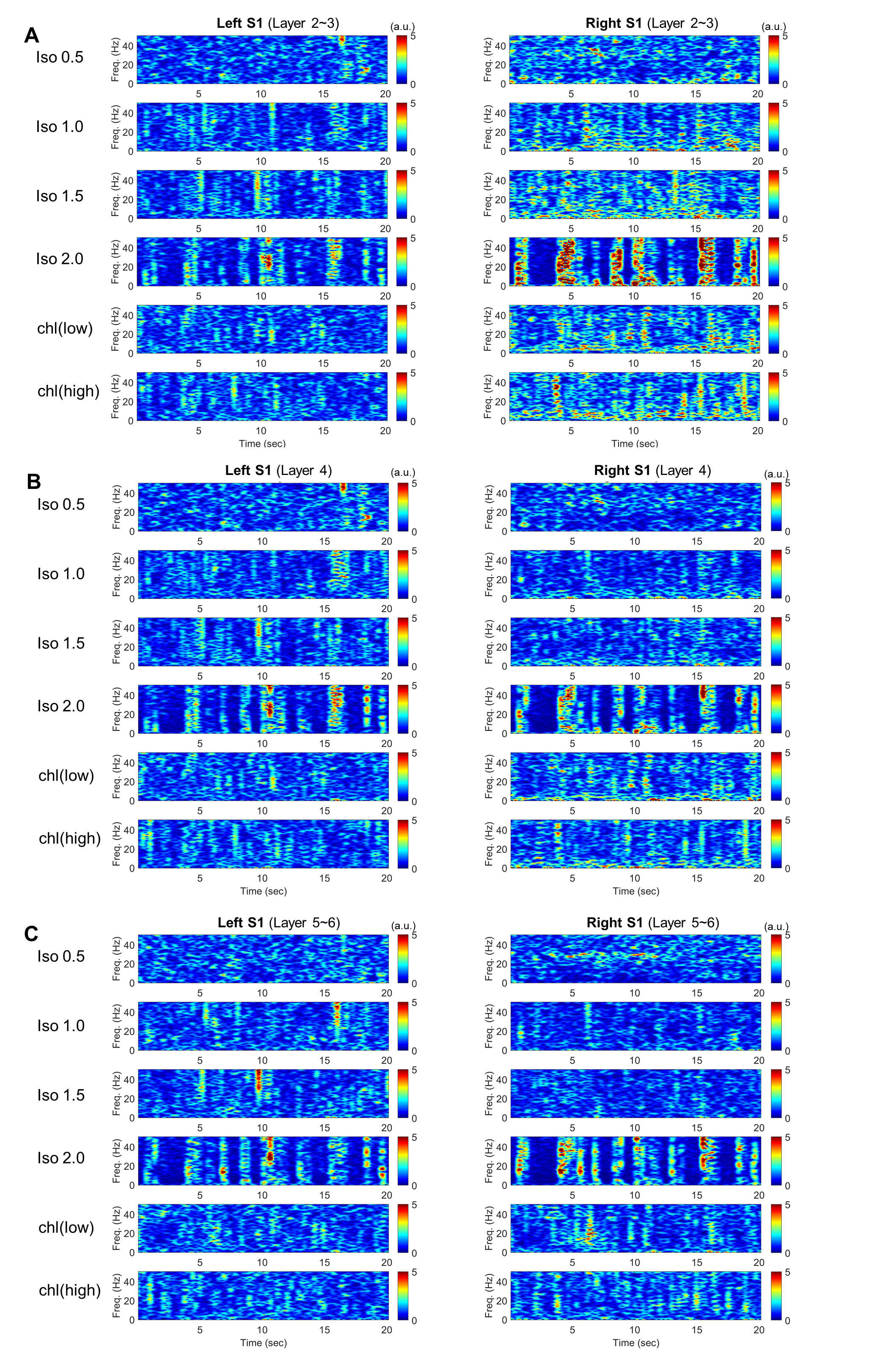
**

**Supplementary Figure 7.** Comparison of the electrode depth across different animals after realignment: LFP and CSD response from individual animals (isoflurane 0.5% condition).


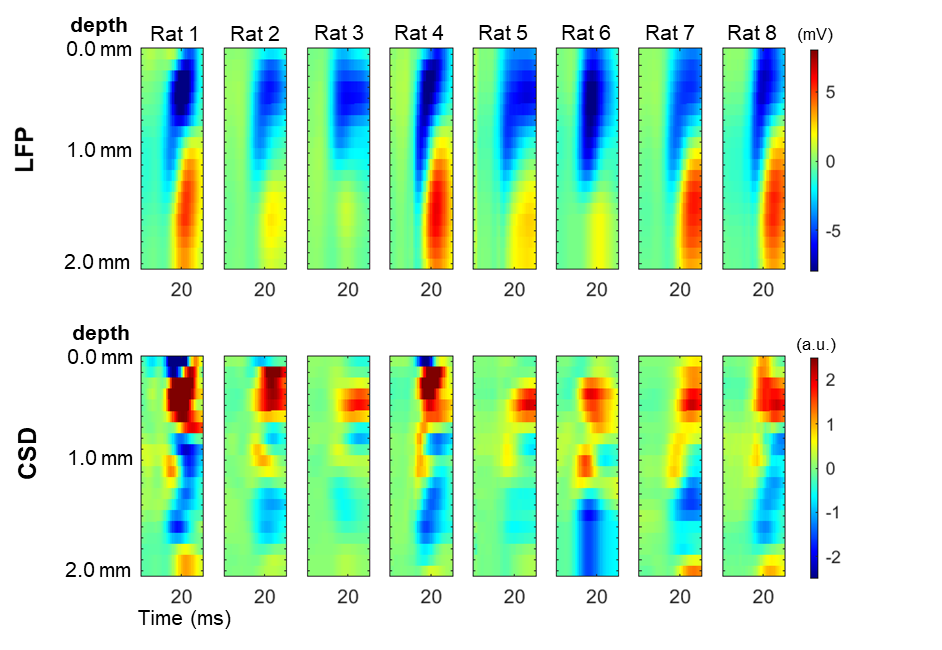

Supplement: Supplementary file 1 — Supplementary Information. [file 41598_2022_13759_MOESM1_ESM.docx]
